# Intraoperative Molecular Profiling of Sentinel Lymph Nodes: Diagnostic Value of Mammaglobin and CK19 in Early Breast Cancer

**DOI:** 10.3390/ijms27104462

**Published:** 2026-05-16

**Authors:** Diana Carolina Zambrano, Andrés Jenuer Matta, María Luisa Maestro de las Casas, Luz Fernanda Sua

**Affiliations:** 1 Center for Basic Sciences, Escuela Nacional del Deporte, Cali 760001, Colombia; 2 Faculty of Basic Sciences, Universidad Santiago de Cali, Cali 760001, Colombia; 3 San Carlos Clinical Hospital, 28001 Madrid, Spain; 4 Valle del Lili Foundation University Hospital, Cali 760001, Colombia

**Keywords:** sentinel lymph node, breast neoplasms, early detection of cancer, polymerase chain reaction

## Abstract

Breast cancer represents a major public health problem worldwide. Despite radical surgery for localized disease, a substantial proportion of patients experience disease recurrence. The aim of this study was to evaluate the expression of the mammaglobin and CK19 genes in sentinel lymph node biopsies from patients with early breast cancer. This descriptive study included 301 sentinel lymph node biopsies from patients with stage I–II breast cancer treated at the San Carlos Clinical Hospital in Madrid, Spain. Metastases were identified using conventional histopathology (H&E), immunohistochemistry (IHC), and molecular detection of mammaglobin and CK19 using PCR-based methods. Associations between variables were assessed using Fisher’s exact test with a 95% confidence level. Statistical analyses were performed using STATA 12.0. The predictive value for metastatic involvement was 12.29% for CK19 and 16.61% for mammaglobin, increasing to 19.27% when conventional staining was combined with immunohistochemistry. The overall sensitivity was 68.9%, and the specificity was 93.42%. Mammaglobin showed slightly better diagnostic performance than CK19, and the combined molecular detection of both genes improved diagnostic accuracy when compared with individual markers. Intraoperative molecular evaluation of sentinel lymph nodes using mammaglobin and CK19 is comparable to conventional histopathological assessment combined with immunohistochemistry. The combined RT-PCR detection of both genes improves diagnostic performance and represents a clinically useful complementary tool for the detection of metastatic involvement in early breast cancer.

## 1. Introduction

Breast neoplasms represent the most common malignant tumors in women, accounting for approximately 24.2% of cancers in this population. According to estimates from the International Agency for Research on Cancer through the GLOBOCAN project, about 2.08 million new cases of breast cancer were diagnosed worldwide in 2018, with higher incidence and mortality reported in developed countries. In the same year, approximately 626,679 deaths were attributed to this disease, representing nearly 15% of cancer-related deaths among women [1].

Despite substantial progress in prevention strategies, early detection programs, and adjuvant therapies, a proportion of patients continue to develop metastatic disease, either at the time of initial diagnosis or during disease progression [2].

Multiple risk factors have been associated with breast cancer development, including female sex, early menarche, late menopause, advanced age at first pregnancy, prolonged hormone replacement therapy, benign proliferative breast diseases, increased mammographic breast density, and germline mutations in susceptibility genes such as BRCA1 and BRCA2. In addition, the identification of prognostic and predictive factors—such as patient age, tumor size, nuclear and histological grade, number of metastatic axillary lymph nodes, hormone receptor expression, and human epidermal growth factor receptor type 2—has enabled the stratification of patients into clinically relevant groups to guide therapeutic decision-making and treatment intensity [3].

The sentinel lymph node is defined as the first lymphatic drainage node of the breast [4]. Selective sentinel lymph node biopsy has become the standard procedure for axillary staging in breast cancer and allows the identification of patients in whom axillary lymphadenectomy offers no additional therapeutic benefit but may cause significant morbidity [5]. Nevertheless, lymphatic metastasis remains one of the most important predictive factors in breast cancer progression. Advances in sentinel node techniques have improved the detection of metastatic involvement and contributed to more accurate disease staging.

Metastasis and tumor invasion constitute the main causes of morbidity and mortality associated with breast cancer. Consequently, the development of molecular techniques capable of validating biomarkers for the early detection of axillary metastatic behavior has become an important focus of research [6,7,8].

In recent years, conceptual changes derived from clinical trials and observational studies have generated considerable variability in therapeutic approaches for patients with metastatic involvement of the sentinel lymph node. This variability highlights the need for molecular diagnostic methods that are rapid, reliable, and highly sensitive and specific. Such approaches may improve diagnostic accuracy, reduce patient anxiety, avoid unnecessary re-interventions, prevent delays in surgical management when lymphadenectomy is required, and decrease associated healthcare costs. Therefore, the aim of this study was to evaluate the expression of the mammaglobin and cytokeratin 19 (CK-19) genes in sentinel lymph node biopsies from patients with early-stage breast cancer.

## 2. Results

The results are presented in two sections: the first describes marker expression and its association with clinical and pathological characteristics, and the second evaluates the diagnostic performance of the molecular markers, including sensitivity, specificity, and predictive values.

### 2.1. Marker Expression and Correlation with Clinical and Pathological Characteristics

A total of 301 sentinel lymph nodes were obtained from the patients included in this study. Mammaglobin expression was detected in 50 nodes (16.7%). Among these patients, 19 (38%) were older than 60 years, eight (16%) had a family history of breast cancer, and seven (14%) had a previous oncological history. In addition, 15 patients (30%) in the G0P0 reproductive group were positive for mammaglobin. Prophylactic surgery was reported in 49 patients (98%), while only 2% had a confirmed genetic history (Table 1). No statistically significant association was identified between clinical characteristics and the expression of mammaglobin or CK19 (Table 1).

Cytokeratin 19 (CK19) expression was identified in 37 sentinel nodes (12.3%). Among these patients, five (13.5%) had a family history of breast cancer, nine (24.3%) had a previous oncological history, and 10 (27%) belonged to the G0P0 reproductive group. Similarly, no statistically significant association was observed between the evaluated clinical characteristics and CK19 expression (Table 1).

Regarding immunohistochemical and pathological features, among the mammaglobin-positive nodes, 37 (74%) expressed estrogen receptors and 36 (72%) expressed progesterone receptors. The Ki-67 ≤ 5% marker was present in 25 cases (50%), and 41 cases (82%) were negative for human epidermal growth factor receptor 2 (HER2). In the pathological assessment, 28 nodes (56%) were positive by hematoxylin and eosin staining (H&E), nine (18%) were positive by immunohistochemistry (IHC), and 37 (74%) were positive when both methods were combined (H&E + IHC).

Among the CK19-positive nodes, 26 (70.3%) expressed estrogen receptors and 27 (73%) expressed progesterone receptors. The Ki-67 ≤ 14% marker was present in 19 cases (51.4%), and 32 cases (86.5%) were negative for HER2. In addition, 20 nodes (54.1%) were positive by H&E staining, eight (21.6%) were positive by IHC, and 28 (75.7%) were positive using the combined H&E + IHC approach.

A statistically significant association was observed between mammaglobin and CK19 positivity and the pathological detection methods H&E, IHC, and H&E + IHC (Table 2).

### 2.2. Diagnostic Performance: Sensitivity and Specificity of Molecular Markers

The diagnostic performance of mammaglobin, CK19, and their combined evaluation was analyzed using histopathological examination as the reference standard. Using H&E as the reference method, mammaglobin showed a sensitivity of 59.57%, while CK19 showed a sensitivity of 42.55%. The combined detection of mammaglobin and CK19 increased the sensitivity to 61.7%. The specificity values were 91.34% for mammaglobin, 93.31% for CK19, and 89.37% for the combined markers (Table 3).

When the combined H&E + IHC method was used as the reference standard, mammaglobin showed a sensitivity of 63.79%, CK19 demonstrated a sensitivity of 48.28%, and the combined mammaglobin + CK19 approach reached a sensitivity of 68.97%. The corresponding specificity values were 94.65% for mammaglobin, 96.3% for CK19, and 93.42% for the combined markers (Table 4).

Overall, the combined evaluation of mammaglobin and CK19 showed improved diagnostic sensitivity compared with each marker individually, while maintaining high specificity when histopathological methods (H&E and H&E + IHC) were used as reference standards. These findings suggest that the combined molecular assessment may enhance the detection of metastatic involvement in sentinel lymph nodes compared with single-marker analysis.

## 3. Discussion

Breast cancer is a systemic disease that generates a major health, social and economic problem; it is the most frequently diagnosed neoplasm in Western women, and tumor characteristics are classified and associated with age [9].

Selective sentinel lymph node biopsy is the procedure of choice for axillary staging of breast cancer and allows the selection of a subgroup of patients in whom axillary lymphadenectomy does not provide any additional benefit but does provide undesirable side effects [10]. In recent years, the conceptual changes introduced by clinical trials and observational studies have produced a great disparity of criteria regarding the therapeutic approach to the diagnosis of the metastatic sentinel node. The state of the lymph nodes is the most important prognostic indicator in breast cancer and directly affects the clinical management of patients, traditionally performed on the basis of their staging. It leads to complete axillary lymphadenectomy, which produces high morbidity in the medium and long term. However, in early tumors up to 3 cm, there are no lymphatic metastases in 68% to 97% of patients. Radio-guided surgery of the sentinel node allows the analysis of the first lymph node that receives tumor drainage; if it is negative, lymphadenectomy is avoided. Considering these advantages of the procedure, the objective of this research was to evaluate the expression of the mammaglobin and CK-19 genes in sentinel node biopsies in early breast cancer, with the purpose of developing a molecular methodology that allows the accurate diagnosis of metastases to this node and can be used in the same surgical act, avoiding reinterventions.

Our research corroborates that the molecular technique by Polymerase Chain Reaction (PCR and qPCR) is safe, with sensitivity, specificity, PPV and NPV comparable to those of conventional techniques: a sensitivity of 68.9%, specificity of 93.42%, PPV of 71.43% and NPV of 92.65%.

Several limitations of this study should be considered when interpreting the results. First, the sensitivity of the molecular detection strategy may be influenced by the presence of tumor subtypes with low or absent CK19 expression. Although CK19 is widely used as a molecular marker for epithelial tumor cells, certain histological variants of breast cancer may show reduced or negative CK19 expression, which could lead to underestimation of metastatic involvement when this marker is used alone. This biological variability may partially explain the moderate sensitivity observed in our series and highlights the importance of combining molecular markers, such as mammaglobin and CK19, to improve diagnostic performance.

In addition, technical factors related to intraoperative tissue handling may also affect sensitivity. The use of frozen tissue fragments implies the possibility that the analyzed portion does not contain metastatic tumor cells, particularly in cases of micrometastasis or isolated tumor cells. Similarly, methodological variables related to primer design and amplification efficiency in PCR assays may influence the detection threshold.

Although this study included 301 sentinel lymph nodes, which represents a substantial sample for evaluating diagnostic performance, the sample size may still be insufficient to draw definitive conclusions regarding long-term survival outcomes or detailed subgroup analyses according to tumor molecular subtypes or histological variants. Therefore, larger prospective studies with extended follow-up are necessary to confirm the potential prognostic implications of molecular detection of metastasis in sentinel lymph nodes.

The aim of having a reliable and accurate preoperative diagnosis lies in performing less aggressive surgeries and reducing the high morbidity generated by radical breast treatments. Currently, the molecular technique called one-step nucleic amplification (OSNA by SysmexTM) with RT-LAMP (Reverse Transcriptase Loop-mediated Isothermal Amplification) is considered the procedure of choice for pathological study of the sentinel node, resulting in greater efficiency for the health system, with sensitivity values between 87.5% and 98.1% and specificity between 90.8% and 94.3% [11,12]. However, the greatest barrier for conventional pathologists is the lack of material for further study, which would not be necessary according to the evidence presented [13,14].

Molecular management by intraoperative qPCR minimizes and standardizes the histological evaluation of the sentinel node, avoiding inter- and intraobserver dependence in the intraoperative cytological study. Different studies have been carried out, including the detection of biomarkers such as mammaglobin and CK 19 using molecular and histopathological techniques (H&E + IHC) to establish a comparison with intraoperative detection of metastases in the sentinel node. The sensitivity obtained was 92% with 97% specificity for RT-PCR [15,16,17].

It should be noted that, when we compared the sensitivity and specificity of CK19 alone versus mammaglobin, mammaglobin in our study surpassed CK19 in the positive diagnosis according to the test as follows: CK19 12.29% vs mammaglobin 16.61%. Combining the two genes, we had 18.6%, surpassing hematoxylin and eosin alone at 15.61% and being comparable with H&E + IHC at 19.27%. In molecular studies, it has been seen that the level of expression of CK19 mRNA correlates with the volume of metastasis, which was also evidenced in our work with CK19 and mammaglobin [18].

All our cases were imprinted before being fractionated for molecular study, which is especially recommended to avoid contamination and correlate with the result, thus avoiding false negatives when a molecular technique is used. This allows for evidence that microscopic observation and cytological correlation is the basis of molecular pathology. Some recommend the molecular technique as the first option for pathological study, and if it is not available, study by freezing the lymph node is acceptable. This is increasingly applied in specialized centers for breast cancer in Spain and could in the future be used in a center such as the Valle del Lili Foundation in Colombia. The Breast Unit of each hospital must provide the quality of the mRNA of the lymph node tissue and optimize the response time by the molecular pathology laboratory when this type of diagnostic methodology is established.

## 4. Materials and Methods

### 4.1. Study Type and Study Population

A longitudinal descriptive study was carried out during the period between October 2004 and March 2010, in which 301 female patients between 25 and 65 years of age from the San Carlos Clinical Hospital of Madrid (HCSCM) were included. The studied patients were diagnosed with breast cancer in stages I and II (up to 3 cm) and were candidates for selective biopsy of the sentinel node. The classification of patients was based on the international TNM (Tumor–Node–Metastasis) system. Patients in whom the sentinel node technique was not satisfactory, the portable probe failed, or there were more than 3 sentinel nodes were not included.

### 4.2. Clinical Methodology

#### 4.2.1. Preoperative Stage

The day prior to surgery (18 to 24 h), the patient was injected with 0.2 mL of nanocolloids labeled with Technetium-99m (99mTc) in the nuclear medicine service, with a dose of 0.1 mCi of 99mTc in the periareolar and intradermal region; then a verification scintigraphy was performed, verifying the migration of the colloid to the armpit and the count of the number of sentinel nodes by means of SPECT-CT.

#### 4.2.2. Operative Stage

An intraparenchymal puncture was performed in the superior external quadrant of the breast; 1–3 mL of isosulfan blue (patent blue or methylene blue) was used and massaged for 10 min; and with a portable probe the sentinel node was located for the counting and comparison of the injection points of the marking. Subsequently, the sentinel node was removed.

#### 4.2.3. Analytical Methodology

The sentinel nodes were divided into one or two fractions, the first of them for histopathological analysis following standard procedures (freezing, imprinting, H&E staining and IHC) and the second fraction for molecular analysis. These fractions were added to guanidine solution (RNA STAT-60 Tel-Test, Inc., Friendswood, TX, USA) and stored at minus 80 °C in the biobank.

#### 4.2.4. Molecular Biology Technique

RNA extraction was performed using the protocol described for the RNeasy Mini Kit (Qiagen, Hilden, Germany). The amplification of the mammaglobin and *CK19* genes was performed by RT-PCR, using 5 μL of RNA. The forward primers were 5′ CAA, ACG, GAT, GAA, ACT, CTG, AGC, AAT, GTT, GA and the reverse were 5′ TCT, GTG, AGC, CAA, AGG, TGT, AGA for mammaglobin; the forward primers were 5′ AGA, TGA, GCA, GGT, CCG, AGG, TTA and the reverse were 5′ CCT, GAT, TCT, GCC, GCT, CAC, TAT, CA for CK19. The analysis of the amplified products by PCR was performed on a 12% polyacrylamide gel, using the PBGD porphobilinogen deaminase gene as the amplification control gene.

### 4.3. Statistical Analysis

The statistical analysis was performed with STATA 12.0 (StataCorp, College Station, TX, USA) [19] software; the occurrence and association of mammaglobin and CK19 in the RT-PCR was evaluated with the exact Fisher [20,21,22] test with a significance level of *p* < 0.05. The diagnostic validity indices sensitivity (S), specificity (E), positive predictive value (PPV) and negative predictive value (NPV) were estimated for mammaglobin and CK19 by RT-PCR, taking H&E and the H&E + IHC combination as gold-standard tests [23,24]. Each value was determined with a confidence level of 95%. For the survival analysis for mammaglobin and CK19 by RT-PCR, H&E and the H&E + IHC combination, the Kaplan–Meier estimator was used, and the Log-Rank test was used to evaluate differences between the observed survival curves [25].

### 4.4. Ethical Considerations

This research was approved by the biomedical research ethics committee of the Valle del Lili Foundation, the human ethics committee of the San Carlos Clinical Hospital in Madrid, and the biomedical research ethics committee of the level IV hospital center, registered in Minute No. 079 of 11 April 2018.

## 5. Conclusions

Intraoperative molecular evaluation of the sentinel lymph node using PCR/qPCR to detect mammaglobin and CK19 represents a valid, safe, and clinically useful diagnostic approach for identifying metastases in early breast cancer. This strategy showed high specificity and a high negative predictive value, with diagnostic performance comparable to that of conventional histopathological techniques such as hematoxylin and eosin (H&E) staining and immunohistochemistry (IHC).

Although the overall sensitivity was moderate (68.9%), this may be explained by biological factors (e.g., CK19-negative tumors), technical aspects related to frozen tissue sampling, and methodological variables such as primer design. Nevertheless, mammaglobin demonstrated better diagnostic performance than CK19, and the combined analysis of both markers improved the detection accuracy, reaching values comparable to those for the combined H&E + IHC evaluation.

Importantly, the implementation of intraoperative molecular techniques, including qPCR-based assays or methods such as the one-step nucleic acid amplification assay developed by Sysmex Corporation, enables more accurate axillary staging during the same surgical procedure. This approach can support real-time surgical decision-making, allowing surgeons to determine the need for immediate axillary management, thereby reducing the likelihood of repeat surgeries, avoiding unnecessary lymphadenectomies, and decreasing the morbidity associated with more extensive procedures.

Overall, these findings support the integration of intraoperative molecular pathology into routine clinical practice within specialized Breast Units, provided that adequate RNA preservation, standardized sample collection, and rapid laboratory processing are ensured. Under these conditions, this diagnostic strategy may contribute to more efficient surgical management of early breast cancer and improved patient outcomes, including in referral centers in developing countries.

## Figures and Tables

**Table 1:** Pathological history of the patients in this study.

Characteristic	Mammaglobin Positive (n = 50)		Mammaglobin Negative (n = 251)		*p*-Value	CK19 Positive (n = 37)		CK19 Negative (n = 264)		*p*-Value
	n	%	n	%		n	%	n	%	
**Age (years)**					**0.46**					**0.17**
<30	0	0	3	1.2		1	2.7	2	0.8	
31–40	7	14	16	6.4		6	16.2	17	6.4	
41–50	10	20	57	22.7		8	21.6	59	22.3	
51–60	14	28	72	28.7		10	27	76	28.8	
>60	19	38	103	41		12	32.4	110	41.7	
**Family history**					**0.20**					**0.11**
Breast cancer	8	16	68	27.1		5	13.5	71	26.9	
Other cancers	7	14	40	15.9		9	24.3	38	14.4	
None	35	70	143	57		23	62.2	155	58.7	
**Personal history**					**0.50**					**0.76**
Breast cancer	1	2	16	6.4		1	2.7	16	6.1	
Other cancers	2	4	15	6		1	2.7	16	6.1	
None	47	94	220	87.6		35	94.6	232	87.9	
**Reproductive history**					**0.13**					**0.39**
G0P0	15	30	49	19.5		10	27	54	20.5	
Others	35	70	202	80.5		27	73	210	79.5	
**Prophylactic surgery**					**0.31**					**0.23**
Yes	49	98	250	99.6		36	97.3	263	99.6	
No	1	2	1	0.4		1	2.7	1	0.4	
**BRCA status**					**0.06**					**1.00**
Positive	1	2	0	0		0	0	1	0.4	
Negative	2	4	3	1.2		0	0	5	1.9	
No data	47	94	248	98.8		37	100	258	97.7	

n: absolute frequency, %: relative frequency (%), *p*: Fisher’s test *p*-value.

**Table 2:** Expression of mammaglobin and CK19 according to histopathological status of the sentinel lymph node.

Characteristic	Mammaglobin Positive (n = 50)		Mammaglobin Negative (n = 251)		*p*-Value	CK19 Positive (n = 37)		CK19 Negative (n = 264)		*p*-Value
	n	%	n	%		n	%	n	%	
**Estrogen receptor**					**0.19**					**0.11**
Positive	37	74	209	83.3		26	70.3	220	83.3	
Negative	13	26	39	15.5		11	29.7	41	15.5	
No data	0	0	3	1.2		0	0	3	1.1	
**Progesterone receptor**					**0.76**					**0.79**
Positive	36	72	189	75.3		27	73	198	75	
Negative	14	28	59	23.5		10	27	63	23.9	
No data	0	0	3	1.2		0	0	3	1.1	
**Ki-67 (%)**					**0.08**					**0.16**
≤14	25	50	100	39.8		19	51.4	106	40.2	
15–50	14	28	114	45.4		10	27	118	44.7	
>51	11	22	34	13.5		8	21.6	37	14	
No data	0	0	3	1.2		0	0	3	1.1	
**HER2**					**0.33**					**0.86**
Positive	9	18	28	11.2		5	13.5	32	12.1	
Negative	41	82	220	87.6		32	86.5	229	86.7	
No data	0	0	3	1.2		0	0	3	1.1	
**H&E**					**<0.01**					**<0.01**
Positive	28	56	19	7.6		20	54.1	27	10.2	
Negative	22	44	232	92.4		17	45.9	237	89.8	
**IHC**					**<0.01**					**<0.01**
Positive	9	18	2	0.8		8	21.6	3	1.1	
Negative	12	24	230	91.6		9	24.3	233	88.3	
Not performed	29	58	19	7.6		20	54.1	28	10.6	
**H&E + IHC**					**<0.01**					**<0.01**
Positive	37	74	21	8.4		28	75.7	30	11.4	
Negative	13	26	230	91.6		9	24.3	234	88.6	

n: absolute frequency, %: relative frequency (%), *p*: Fisher’s test *p*-value. Abbreviations: H&E, hematoxylin and eosin staining; IHC, immunohistochemistry; HER2, human epidermal growth factor receptor 2.

**Table 3:** Sensitivity, specificity and predictive values of mammaglobin, CK19 and mammaglobin + CK19 according to H&E.

H&E			
Indicator	Mammaglobin	CK19	Mammaglobin + CK19
	PCR (%)	PCR (%)	(%)
**Diagnostic test**			
Sensitivity	59.57	42.55	61.7
Specificity	91.34	93.31	89.37
Positive predictive value	56	54.05	51.79
Negative predictive value	92.43	89.77	92.65

I.C. 95%: Interval at 95% confidence.

**Table 4:** Sensitivity, specificity and predictive values of CK19 and mammaglobin + CK19 according to H&E.

H&E + IHC			
Indicator	Mammaglobin	CK19	Mammaglobin + CK19
	PCR (%)	PCR (%)	(%)
**Diagnostic test**			
Sensitivity	63.79	48.28	68.97
Specificity	94.65	96.3	93.42
Positive predictive value	74	75.68	71.43
Negative predictive value	91.63	88.64	92.65

I.C. 95%: Interval at 95% confidence.

## Data Availability

Data availability is subject to authorization from the ethics committee. The project’s principal investigator will escalate the request to the committee and, based on the response, share the information in the respective repositories for review.

## Abbreviations

The following abbreviations are used in this manuscript:

CK-19	Cytokeratin 19
STATA	Statistics and Data Analysis
RT-PCR	Reverse Transcription Polymerase Chain Reaction
qPCR	Quantitative PCR
BRCA 1/2	Breast Cancer Gene 1 and 2
HCSCM	San Carlos Clinical Hospital of Madrid
TNM	Tumor–Node–Metastasis
SPECT-CT	Single-Photon Emission Computed Tomography + CT
H&E	Hematoxylin and eosin
IHC	Immunohistochemistry
PCR	Polymerase Chain Reaction
PBGD	Porphobilinogen deaminase
Ki-67	Ki-67 proliferation antigen
HER2	Human epidermal growth factor receptor 2
CNS-positive	Central Nervous System-positive
PPV	Positive predictive value
NPV	Negative predictive value
RT-LAMP	Reverse Transcriptase Loop-mediated Isothermal Amplification
OSNA	One-step nucleic acid amplification
